# Non-optimal ambient temperatures aggravate insecticide toxicity and affect honey bees *Apis mellifera* L. gene regulation

**DOI:** 10.1038/s41598-023-30264-0

**Published:** 2023-03-09

**Authors:** Mohamed Alburaki, Shayne Madella, Steven C. Cook

**Affiliations:** USDA-ARS Bee Research Laboratory, BARC-East Bldg 306, Office 317, Beltsville, MD 20705 USA

**Keywords:** Gene expression analysis, Biologics

## Abstract

In this study, we conducted a transcriptional analysis of five honey bee genes to examine their functional involvement vis-à-vis ambient temperatures and exposure to imidacloprid. In a 15-day cage experiment, three cohorts of one-day-old sister bees emerged in incubators, were distributed into cages, and maintained at three different temperatures (26 °C, 32 °C, 38 °C). Each cohort was fed a protein patty and three concentrations of imidacloprid-tainted sugar (0 ppb, 5 ppb and 20 ppb) ad libitum. Honey bee mortality, syrup and patty consumption were monitored daily over 15 days. Bees were sampled every three days for a total of five time points. RT-qPCR was used to longitudinally assess gene regulation of *Vg*, *mrjp1*, *Rsod*, *AChE-2* and *Trx-1* using RNA extracted from whole bee bodies. Kaplan–Meier models show that bees kept at both non-optimal temperatures (26 °C and 38 °C) were more susceptible to imidacloprid, with significantly higher mortality (*P* < 0.001 and *P* < 0.01, respectively) compared to the control. At 32 °C, no differences in mortality (*P* = 0.3) were recorded among treatments. In both imidacloprid treatment groups and the control, the expression of *Vg* and *mrjp1* was significantly downregulated at 26 °C and 38 °C compared to the optimal temperature of 32 °C, indicating major influence of ambient temperature on the regulation of these genes. Within the ambient temperature groups, both imidacloprid treatments exclusively downregulated *Vg* and *mrjp1* at 26 °C. *AChE-2* and the poorly characterized *Rsod* gene were both consistently upregulated at the highest temperature (38 °C) compared to the ideal temperature (32 °C) in all treatment groups. *Trx-1* showed no effect to both temperature and imidacloprid treatments and was regulated in an age-related manner. Overall, our results indicate that ambient temperatures amplify imidacloprid toxicity and affect honey bee gene regulation.

## Introduction

The honey bee (*Apis mellifera* L.) is a major eusocial insect pollinator^1–3^. Studying honey bees under field conditions is challenging due to the complexity of their behavior and the ecosystems in which they operate^4–7^. Experiments in cages, which offer a more stable and controlled environment, have commonly been used to investigate a wide range of bee stressors, such as pesticide toxicity, diet behavior, disease and pathogen resistance^8–12^.

Honey bee gene regulation is often studied in relation to abiotic stressors^7^, pathogen infection^13^ and honey bee castes and developmental phases^14^. Such investigations have deepened our understanding and contributed to identifying antioxidant and immune genes involved in honey bee response to various stressors^15,16^. The antioxidant system in honey bees is similar to other insects^17^. It includes enzymes such as SOD, GST and Carbonyl reductase that protect cellular components (e.g., lipids and proteins) from the damaging effects of oxidative free radicals. Less well-understood molecules with reported antioxidant functions include thioredoxin (Trx-1) and related superoxide dismutases (Rsod). Honey bees produce several molecules with a range of functions that possess antioxidant capabilities, including vitellogenin (Vg), a glycolipoprotein best described as a yolk protein, and major royal jelly proteins (mrjps)^15,18,19^. Both *mrjp1* and *Vg* are consequential genes for colony well-being due to their direct involvement in brood food production via nurse bees^20^.

Besides pathogen infection, pesticide exposure is another abiotic stressor that impacts the regulation of honey bee antioxidant genes^9,13,21^. Imidacloprid is a broadly used neonicotinoid insecticide in agriculture for pest management control. It has been described as a contributing factor to bee decline, with the ability to impair honey bee performance even at sublethal doses^21–26^. This neonicotinoid molecule has a high agonistic affinity with nicotinic acetylcholine receptors (nAChR), particularly in the tissue of the nervous system^27^. This effect is usually countered by upregulation of the acetylcholine esterase gene (*AChE-2*) in honey bees^6,28,29^. Exposure to imidacloprid can significantly impact honey bees' antioxidant system and gene regulation^30^. In a previous study concerning caging stress and imidacloprid toxicity, two major antioxidant genes, thioredoxin (*Trx-1*) and a poorly characterized gene (*Rsod*) were found to be constantly upregulated in caged bees during a four-week experiment compared to their sister mates living in-hive^9^.

The effects of pesticide exposure on insects can be exacerbated by thermal stress, which impacts the antioxidant system in exposed insects. It is, however, unclear to what extent these two factors intertwine. Honey bees are exceptional social insects that can thermoregulate their hive environment to facilitate brood development and maintain winter bee cluster temperatures between 33 and 35 °C, irrespective of outdoor temperatures or other environmental conditions^31,32^. It has been demonstrated that honey bees can tolerate relatively high temperatures (44.0 ± 0.96 °C) for a short period of time, as measured within the bee-balling defense against the Japanese yellow hornet, *Vespa simillima*^33^. Given the unique and steady thermobiology of honey bees, synergistic effects between ambient temperature and pesticide toxicity may affect honey bee survival. A recent study showed that acute exposure to sublethal doses of imidacloprid and acetamiprid, both neonicotinoid insecticides, resulted in higher thermal tolerance and greater survival rates of bees^34^.

In this study, we closely investigated the effect of ambient temperatures on the toxicity of imidacloprid at sublethal and lethal concentrations. Furthermore, we conducted a longitudinal study characterizing the regulation of five major honey bee genes (*Vg*, *mrjp1*, *AChE-2*, *Rsod*, *Trx-1*) under two different abiotic stressors; ambient temperatures (26 °C, 32 °C, 38 °C) and exposure to imidacloprid.

## Results

### Nutrient consumption

**Figure 1:**
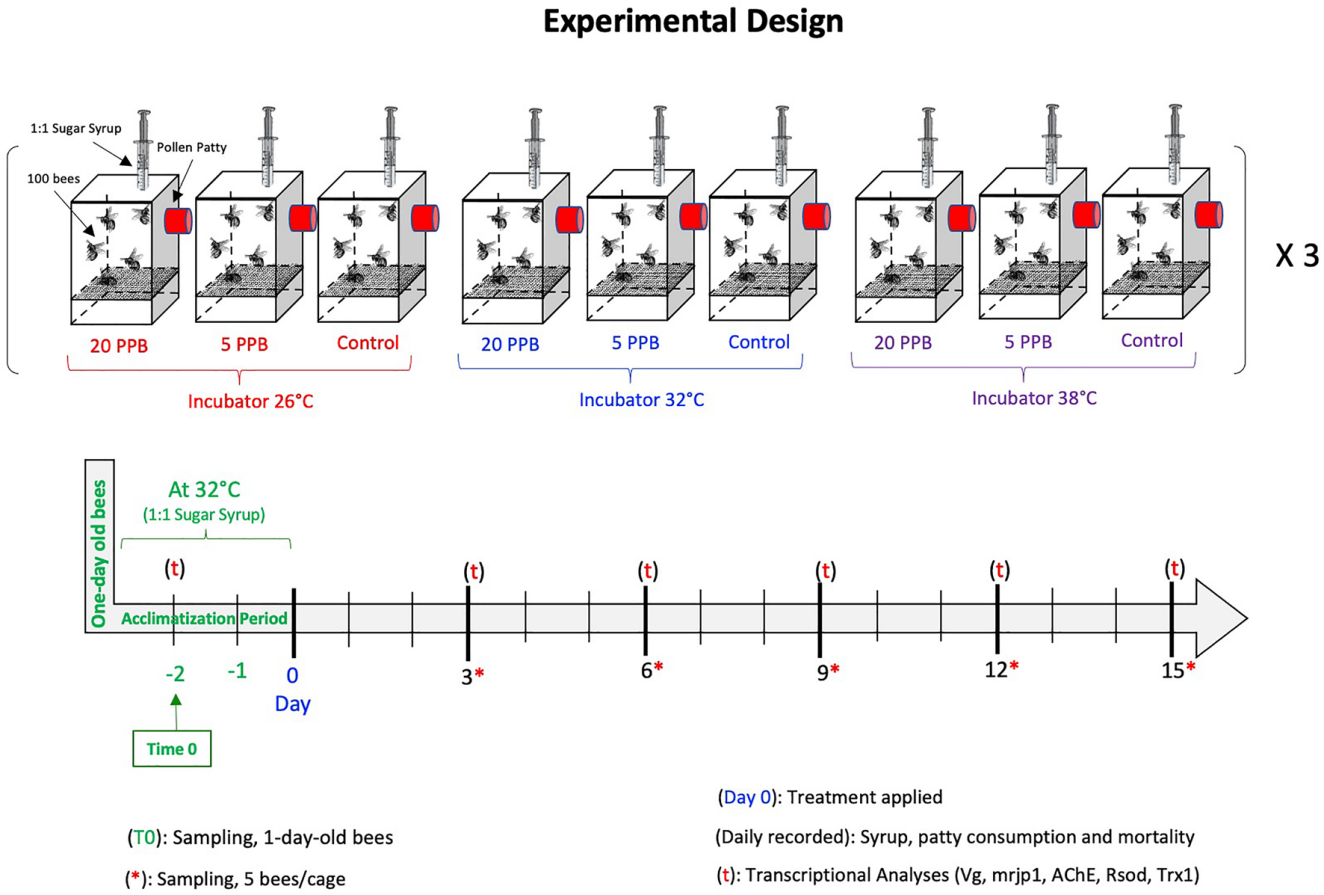
Experimental design and timelines. One-day-old sister bees emerged in an incubator and were distributed into 27 cages (100 bees/cage). Imidacloprid treatments (5 ppb, 20 ppb) were administered through sugar syrup in triplicates. Caged bees were subjected to three different temperatures (26 °C, 32 °C, 38 °C). Bees were sampled five times throughout the 15-day experiment in addition to Time 0 sampling conducted on Day -2.

**Figure 2:**
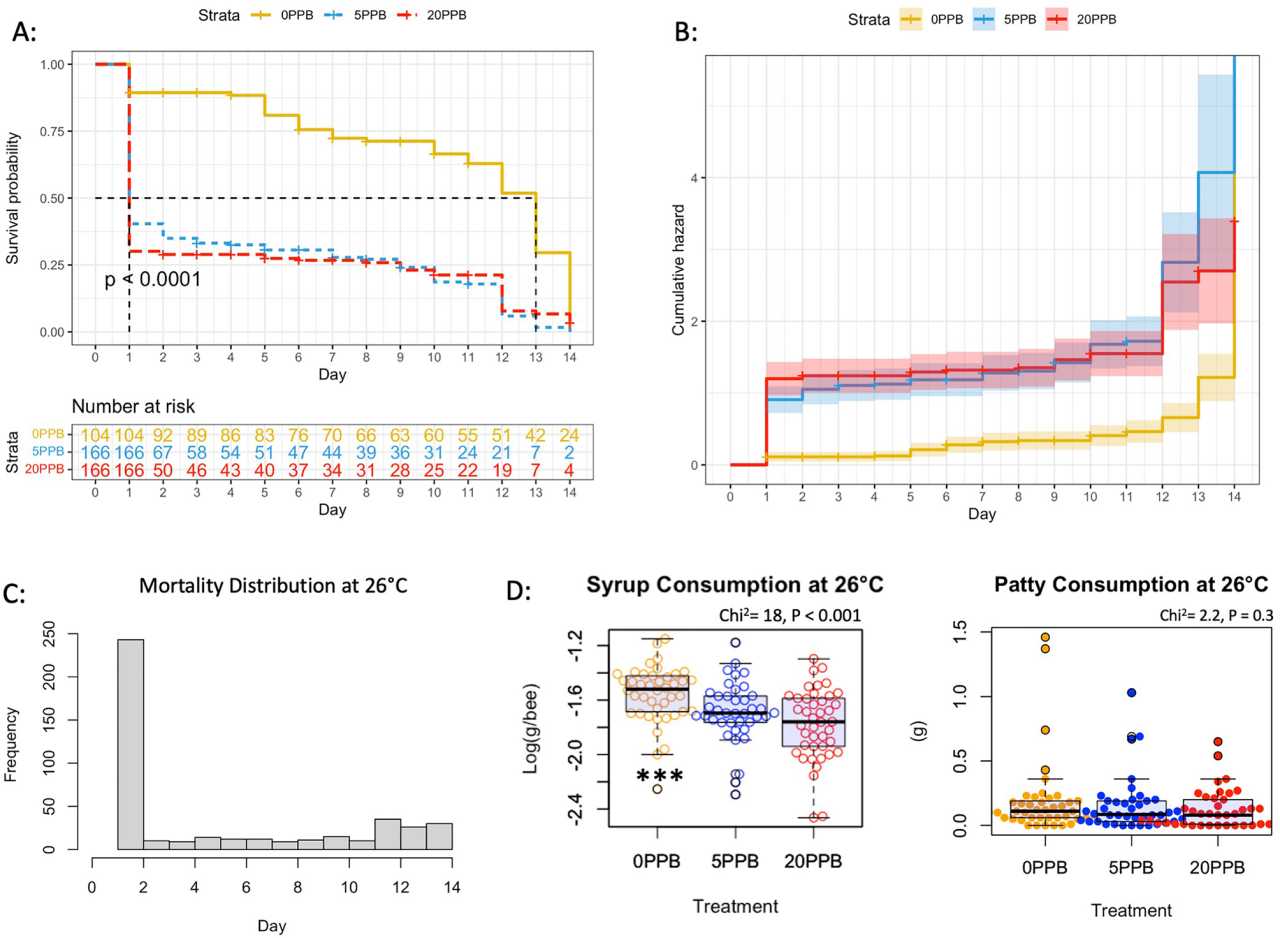
Kaplan–Meier survival probability (**A**) and cumulative hazard (**B**) models conducted on the bee group subjected to 26 °C. The number of bees at risk is estimated through the same model for each treatment category (0 ppb, 5 ppb and 20 ppb). Distribution of overall mortality (**C**) throughout the experiment, average syrup and patty consumptions (**D**) per bee are also given.

**Figure 3:**
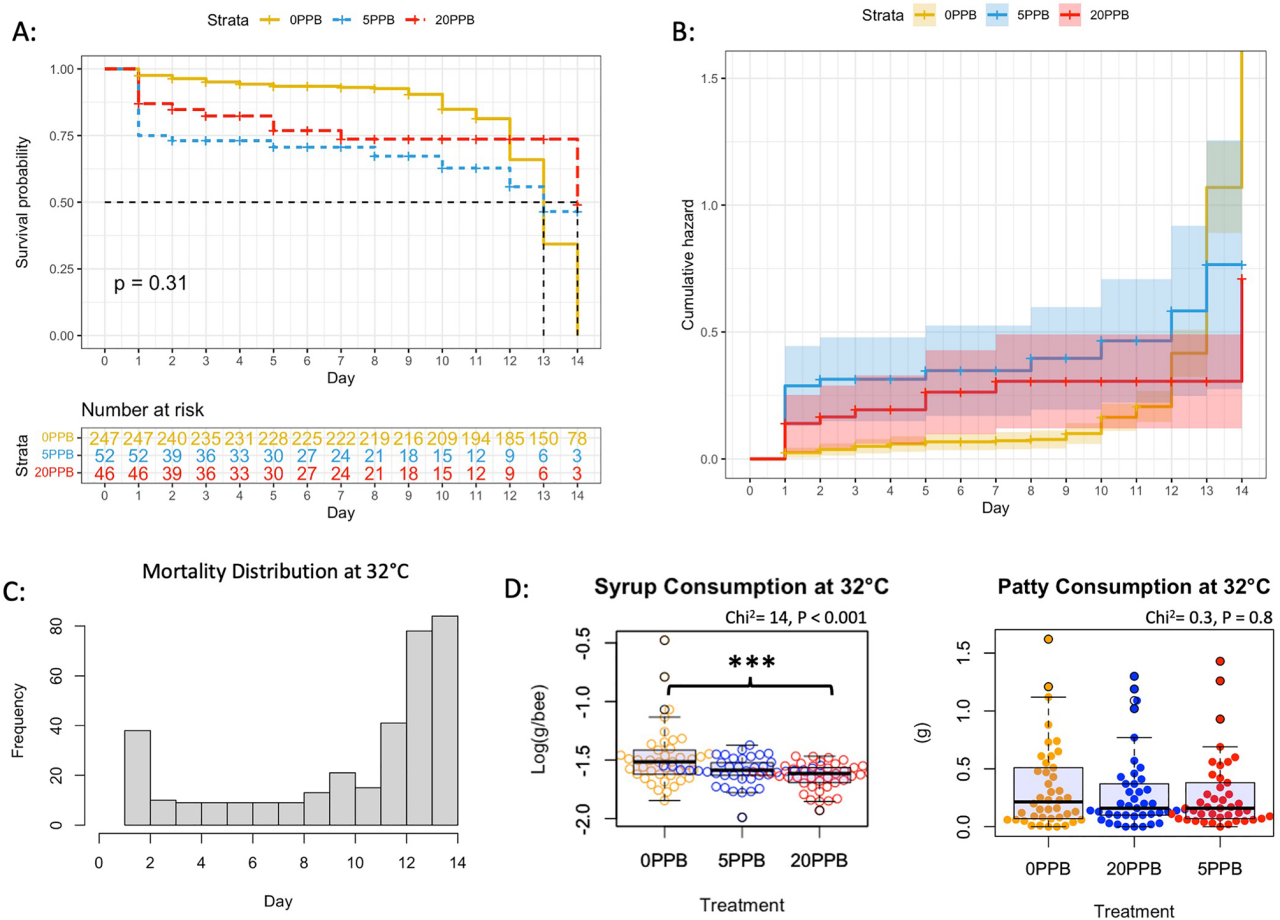
Kaplan–Meier survival probability (**A**) and cumulative hazard (**B**) models conducted on the bee group subjected to 32 °C. The number of bees at risk is estimated through the same model for each treatment category (0 ppb, 5 ppb and 20 ppb). Distribution of overall mortality (**C**) throughout the experiment, average syrup and patty consumptions (**D**) per bee are also given.

**Figure 4:**
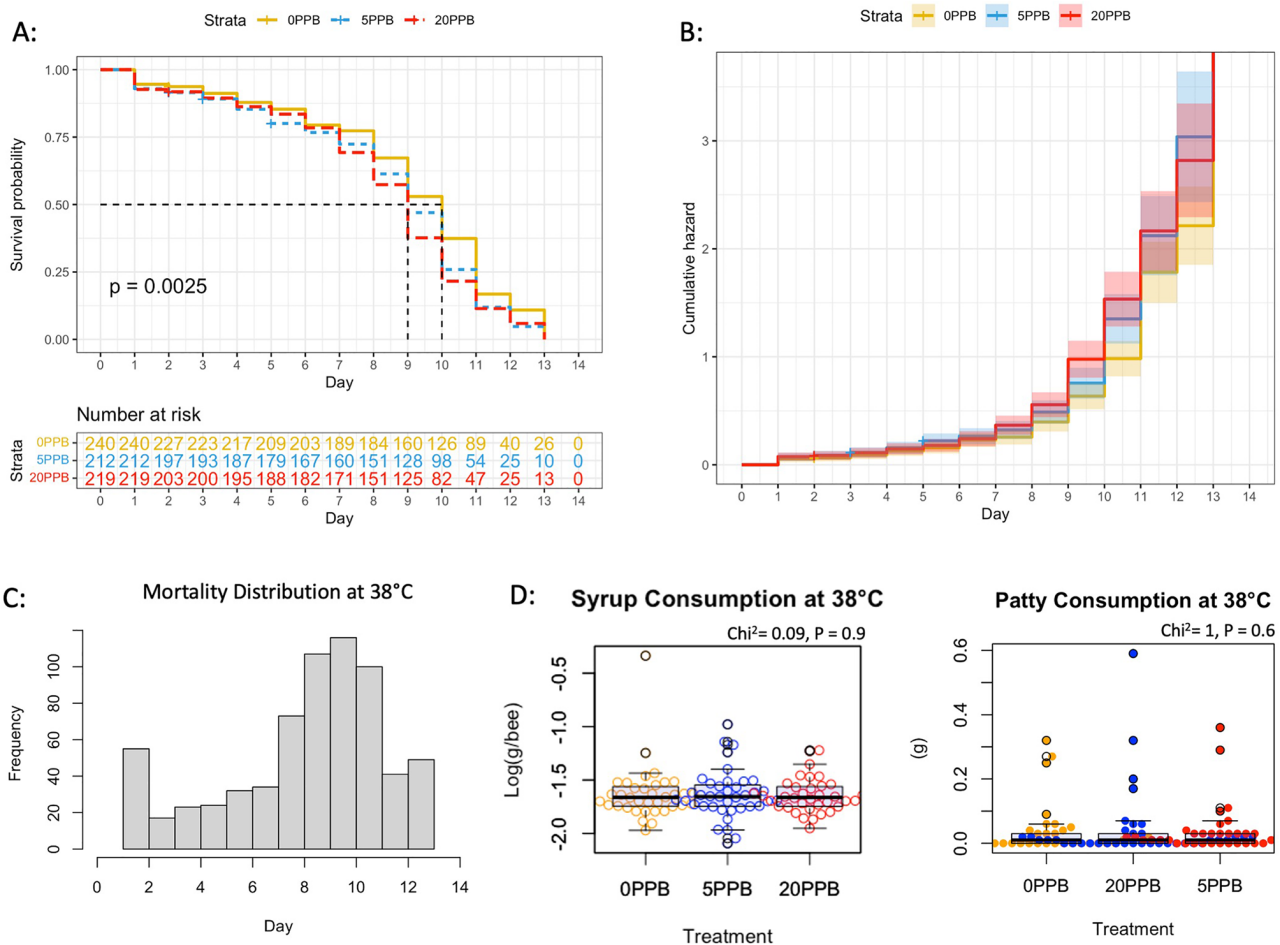
Kaplan–Meier survival probability (**A**) and cumulative hazard (**B**) models conducted on the bee group subjected to 38 °C. The number of bees at risk is estimated through the same model for each treatment category (0 ppb, 5 ppb and 20 ppb). Distribution of overall mortality (**C**) throughout the experiment, average syrup and patty consumptions (**D**) per bee are also given.

When comparing syrup intake across imidacloprid treatments (Fig. 1) within the cage groups subjected to 26 °C, control bees (0 ppb) consumed significantly (*P* < 0.001) more syrup compared to bees exposed to 5 and 20 ppb, Fig. 2. At 32 °C, control bees (0 ppb) consumed significantly (*P* < 0.001) more syrup than bees fed 20 ppb with no differences with bees fed 5 ppb, Fig. 3. No differences (*P* = 0.9) in syrup intake were recorded among imidacloprid treatments for bees kept at 38 °C, Fig. 4. When comparing syrup intake across temperature treatments, our data showed that bees at 32 °C consumed significantly (*P* < 0.001) more syrup than those at 26 °C and 38 °C, Fig. S1. Concerning protein consumption, no differences in consumption were recorded within each temperature category among imidacloprid treatments, Figs. 2, 3, 4. However, across temperature treatments, bees subjected to 38 °C consumed significantly (*P* < 0.001) less patty than those at 26 °C and 32 °C, Fig. S1.

### Bee toxicity and mortality

In the 26 °C category, the Kaplan–Meier model shows significantly (*P* < 0.001) higher toxicity and mortality in bees fed imidacloprid (5 and 20 ppb) compared to the control (0 ppb), Fig. 2A. Acute toxicity was manifested around 24 h of administrating the treatment as seen in the cumulative hazard graph of Fig. 2B. Similar findings (*P* = 0.002) were obtained for bees kept at 38 °C exhibiting more chronic than acute toxicity seen at 26 °C, Fig. 4. However, at the optimal temperature of 32 °C, no significant differences (*P* = 0.31) in bee mortality were observed among the imidacloprid treatments (5 and 20 ppb) and the control groups, Fig. 3.

### Gene regulation

#### Effect of the temperature

**Figure 5:**
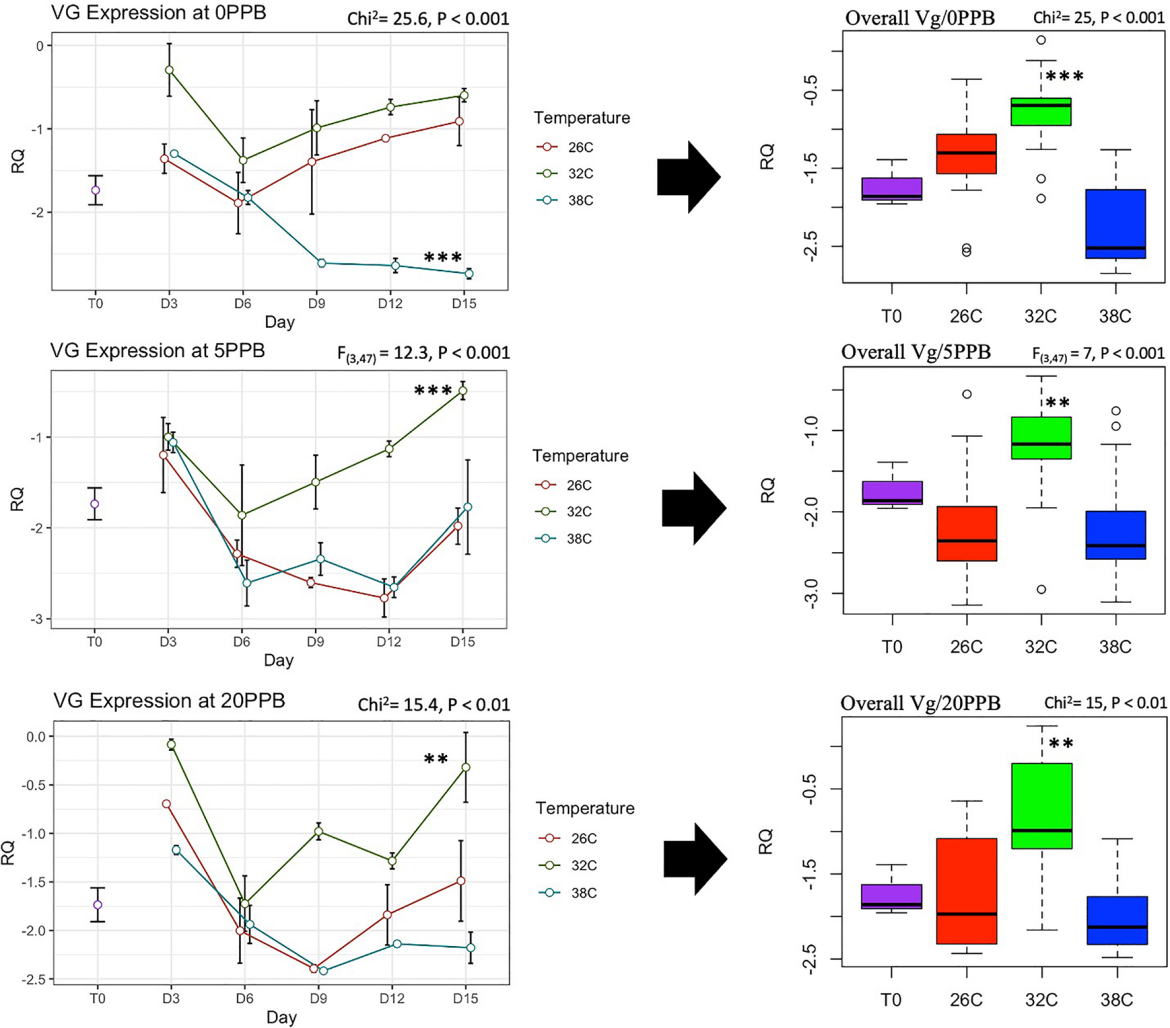
Longitudinal and overall gene expression of *Vg* across imidacloprid (0 ppb, 5 ppb, 20 ppb) and temperature (26 °C, 32 °C, 38 °C) treatments. (T_0_) is the *Vg* expression in newly emerged bees of one-day-old. ANOVA or Kruskal–Wallis’ levels of significance are *P* < 0.05*, *P* < 0.001**, *P* < 0.001***. Error bars in the line graphs are the Standard Error SE.

**Figure 6:**
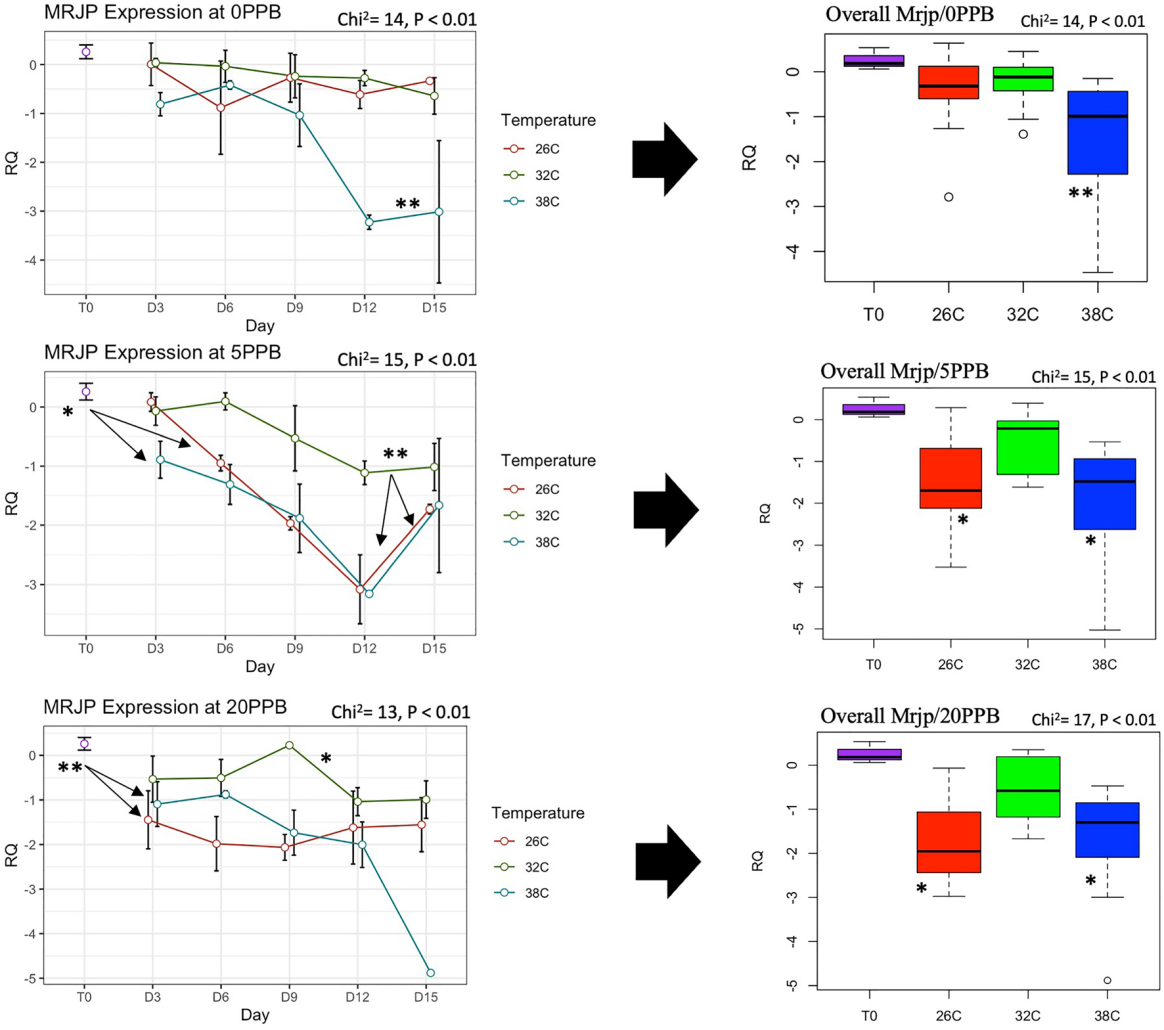
Longitudinal and overall gene expression of *mrjp1* across imidacloprid (0 ppb, 5 ppb, 20 ppb) and temperature (26 °C, 32 °C, 38 °C) treatments. (T_0_) is the *mrjp1* expression in newly emerged bees of one-day-old. Kruskal–Wallis’ levels of significance are *P* < 0.05*, *P* < 0.001**, *P* < 0.001***. Error bars in the line graphs are the Standard Error SE.

**Figure 7:**
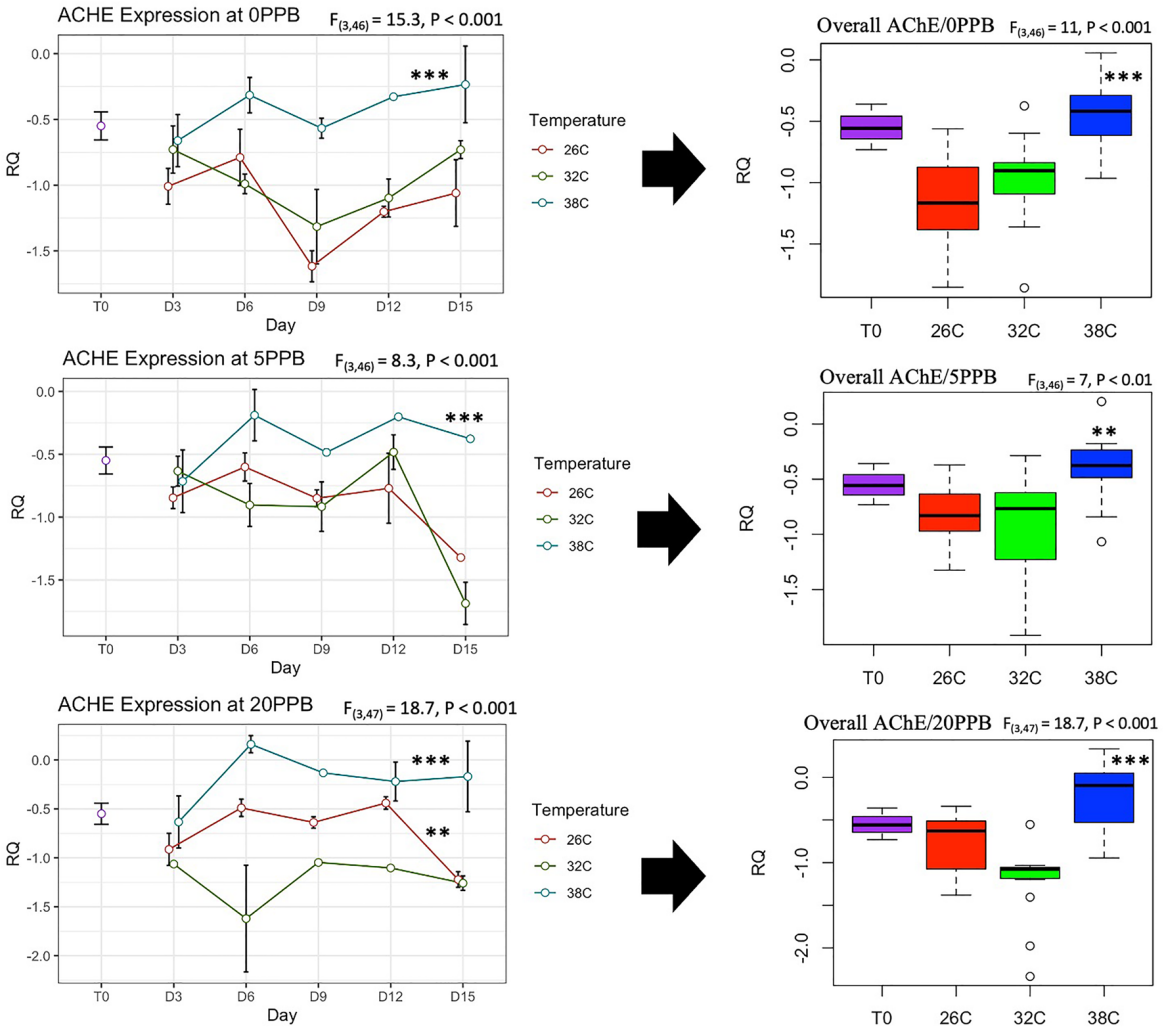
Longitudinal and overall gene expression of *AChE-2* across imidacloprid (0 ppb, 5 ppb, 20 ppb) and temperature (26 °C, 32 °C, 38 °C) treatments. (T_0_) is the *AChE-2* expression in newly emerged bees of one-day-old. ANOVA levels of significance are *P* < 0.05*, *P* < 0.001**, *P* < 0.001***. Error bars in the line graphs are the Standard Error SE.

**Figure 8:**
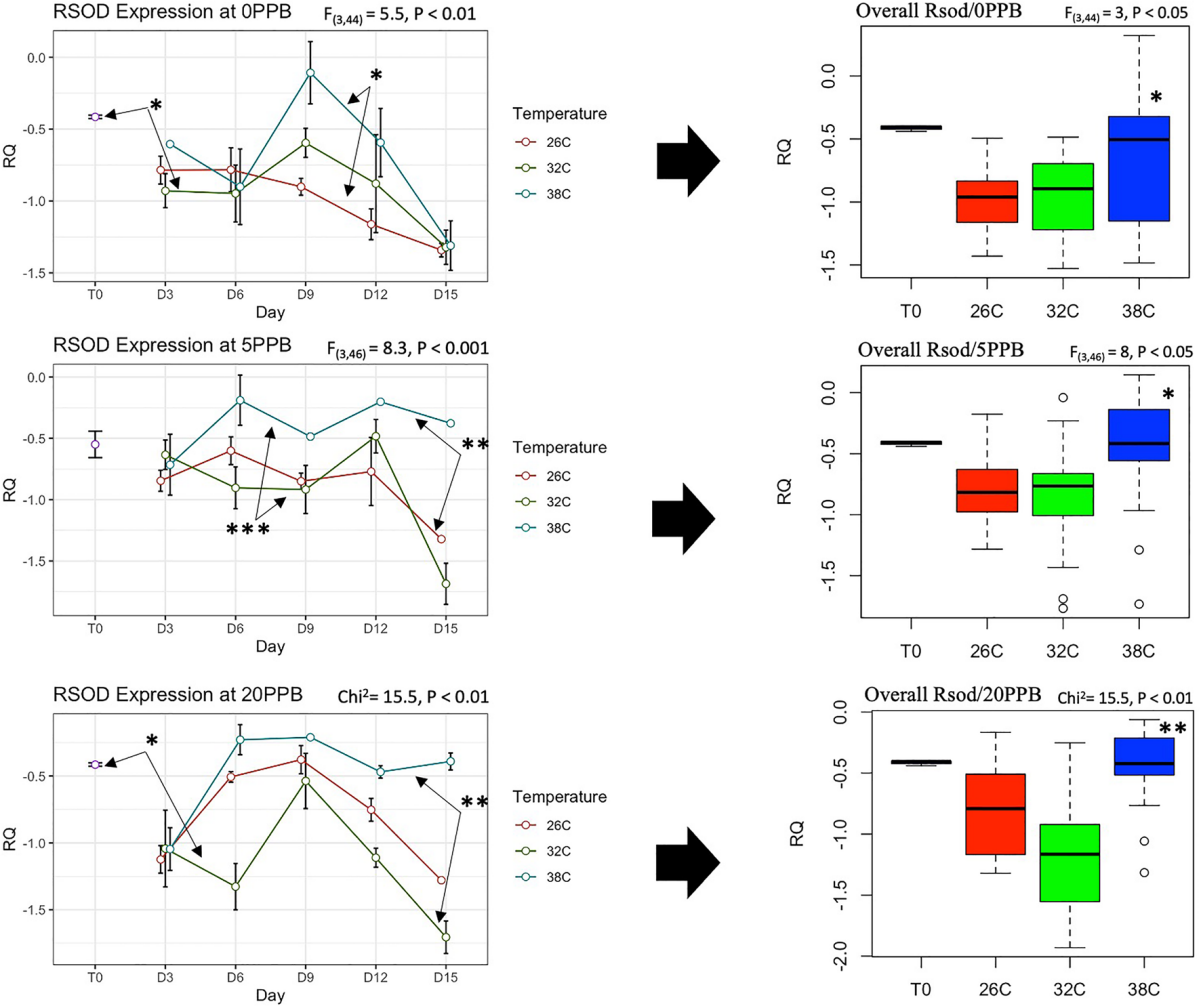
Longitudinal and overall gene expression of *Rsod* across imidacloprid (0 ppb, 5 ppb, 20 ppb) and temperature (26 °C, 32 °C, 38 °C) treatments. (T_0_) is the *Rsod* expression in newly emerged bees of one-day-old. ANOVA or Kruskal–Wallis’ levels of significance are *P* < 0.05*, *P* < 0.001**, *P* < 0.001***. Error bars in the line graphs are the Standard Error SE.

**Figure 9:**
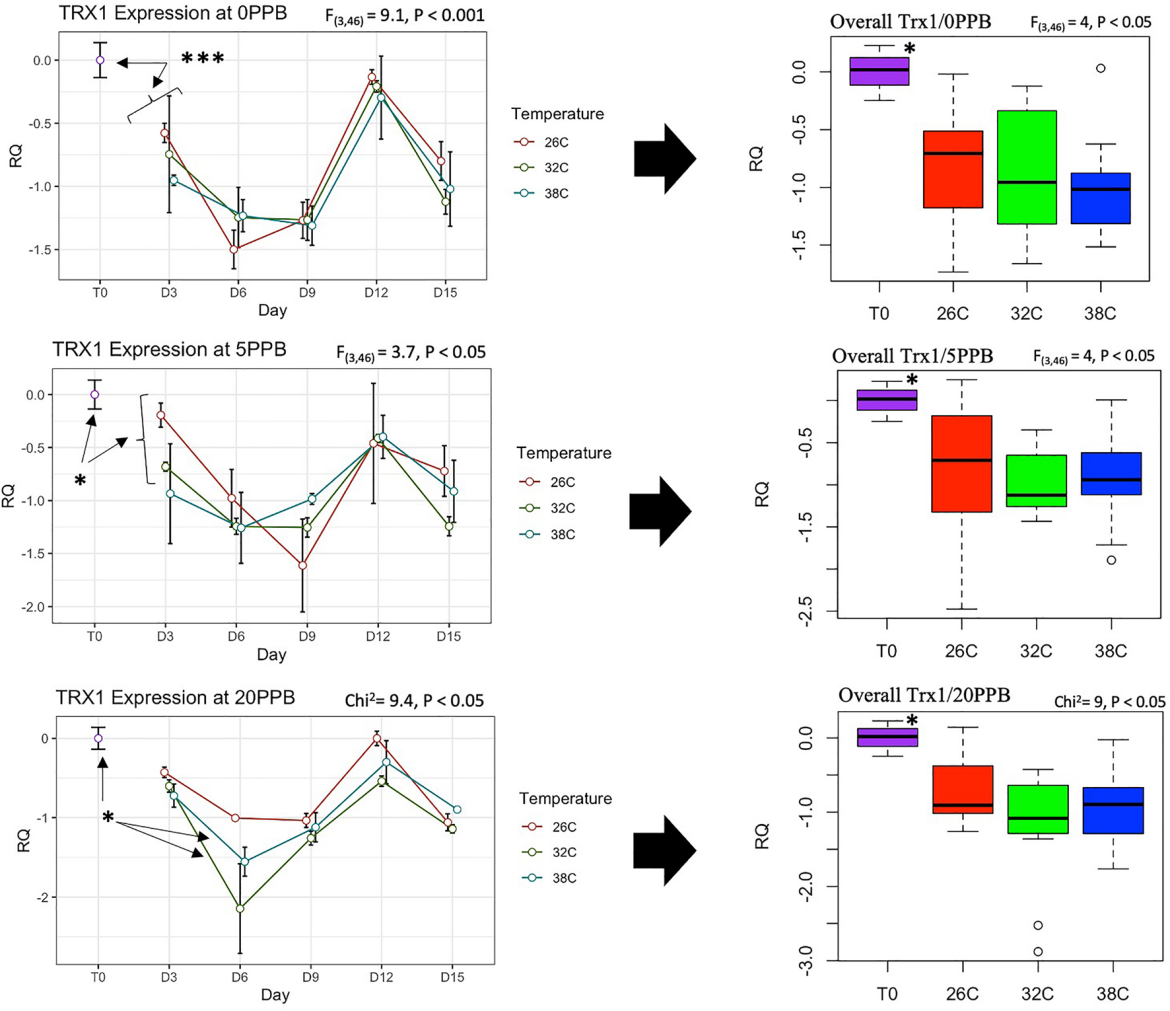
Longitudinal and overall gene expression of *Trx-1* across imidacloprid (0 ppb, 5 ppb, 20 ppb) and temperature (26 °C, 32 °C, 38 °C) treatments. (T_0_) is the *Trx-1* expression in newly emerged bees of one-day-old. ANOVA or Kruskal–Wallis’ levels of significance are *P* < 0.05*, *P* < 0.001**, *P* < 0.001***. Error bars in the line graphs are the Standard Error SE.

The expression of *Vg* was constantly and significantly (*P* < 0.001) upregulated in bees kept at the optimal temperature of 32 °C compared to bees kept at 26 °C and 38 °C, regardless of the imidacloprid treatments, Fig. 5. Moreover, *Vg* expression significantly increased only in bees kept at 32 °C compared to its initial expression at Time 0, Fig. 5. *Mrjp1* expression followed trends similar to *Vg* and was constantly downregulated (*P* < 0.01) at 26 °C and 38 °C compared to 32 °C, Fig. 6. The highest level of *mrjp1* expression was found at Time 0 and diminished throughout time, irrespective of the treatments. Expression of *AChE-2* was significantly (*P* < 0.001) upregulated at 38 °C compared to 26 °C and 32 °C, regardless of the imidacloprid treatment, Fig. 7. Similar to the *AChE-2*, *Rsod* expression was constantly upregulated at 38 °C compared to 26 °C and 32 °C, irrespective of the imidacloprid treatment as well, Fig. 8. *Trx-1* regulation was not affected by temperature changes at any time point, but was significantly (*P* < 0.05) lower in all temperature and imidacloprid groups compared to its initial expression in bees at Time 0, Fig. 9.

#### Effect of the imidacloprid treatment

No significant differences were found in the *Vg* expression among imidacloprid treatment groups, except at 26 °C. At this temperature, bees in the control group (0 ppb) significantly (*P* < 0.05) upregulated their levels of *Vg* compared to bees fed 5 ppb imidacloprid, Fig. S2. Similarly, *mrjp1* expression differed only at 26 °C, in which the control group (0 ppb) expressed significantly (*P* < 0.01) higher *mrjp1* compared to both the 5 and 20 ppb groups, Fig. S3. Concerning the *AChE-2* regulation, this gene varied only at 26 °C, where it was significantly (*P* < 0.01) upregulated at both 5 and 20 ppb compared to the control, Fig. S4. No other significant differences in the regulation of *AChE-2* were observed. The overall expression of *Rsod* and *Trx-1* showed no statistical differences among imidacloprid treatment groups within any temperature categories, Fig. S5 and S6.

#### Principal component analysis

**Figure 10:**
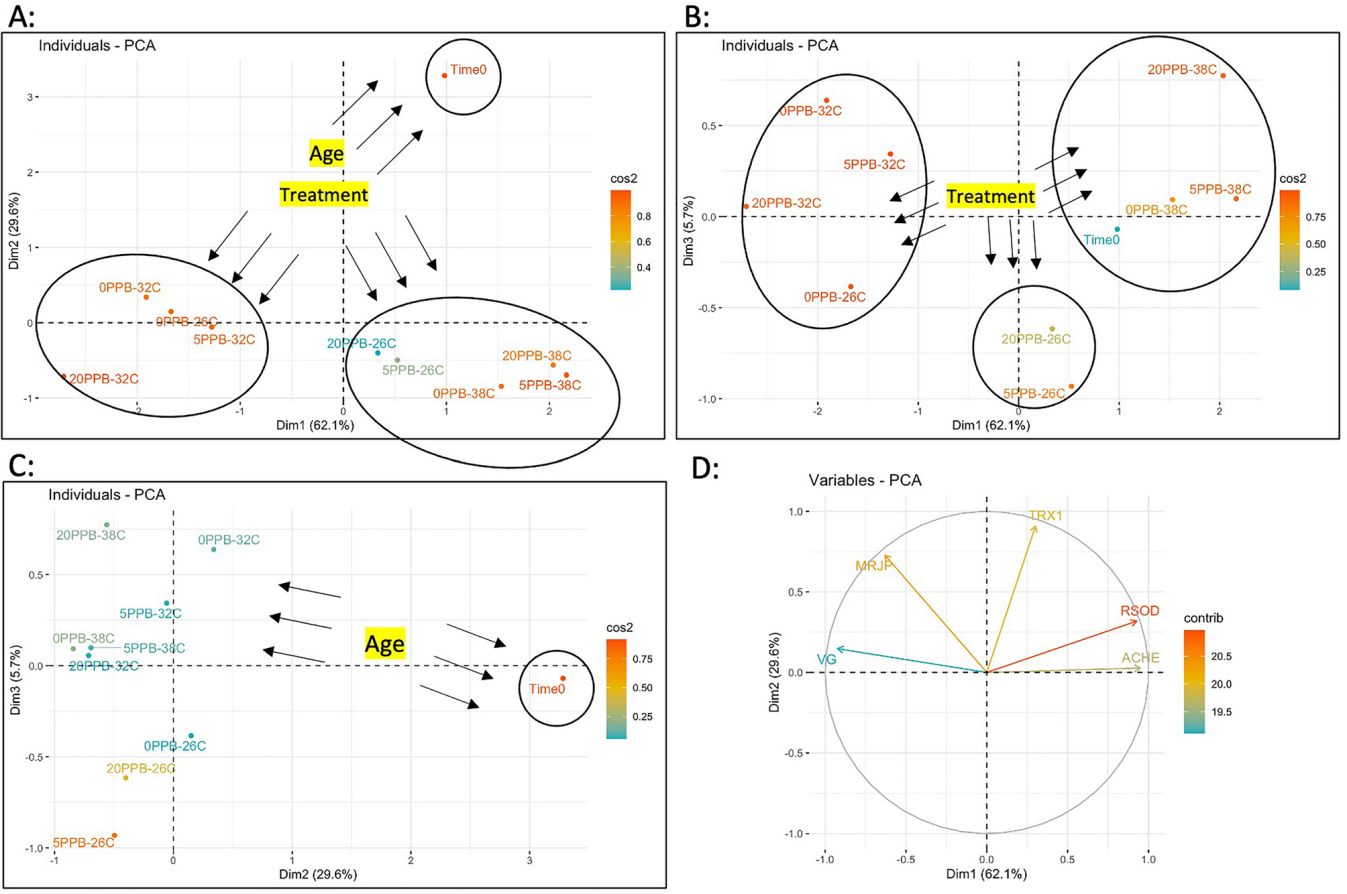
Principal Component Analysis (PCA) conducted on the regulation of the five studied genes (*Vg*, *mrjp1*, *AChE-2*, *Rsod*, *Trx-1*). Percentages of individual variables expressed on components 1 and 2 (**A**), 1 and 3 (**B**), 2 and 3 (**C**) graphically visualized in a 3-dimensional space. Expression and direction of each variable (genes) on Dim1 and 2 (**D**).

The PCA of the gene regulation data of all studied genes revealed that 62.1% of the variables were expressed on Dim1, 29.6% on Dim2 and 5.7% on Dim3, Fig. 10. The PCA discriminates on Dim1 (62.1%) and Dim 2 (29.6%), three major groups that delimited two major factors: 1- “bee age” and 2- “temperature”, Fig. 10A. On Dim1, which explains 62.1% of the variance, two groups of temperature treatments can be observed: 1- bees kept at 32 °C, 2- both bee groups kept at 26 °C and 38 °C, Fig. 10A. Similarly, Dim 1 and 3 grouped the variables mainly based on their respective temperatures, indicating a strong effect of temperature on gene regulation and no visible effect of imidacloprid treatment, Fig. 10B. Dim 2 and 3, however, only delimited the factor “age” by a distinct separation of Time 0 bees from the rest of the groups, Fig. 10C. The PCA displayed by variables showed two sets of genes with opposing function and regulation vis-à-vis the treatments: 1-*Vg* and *mrjp1*, and 2- *Rsod* and *AChE-2*, while *Trx-1* exhibited neutral regulation, Fig. 10D.

#### Gene function and heatmaps

**Figure 11:**
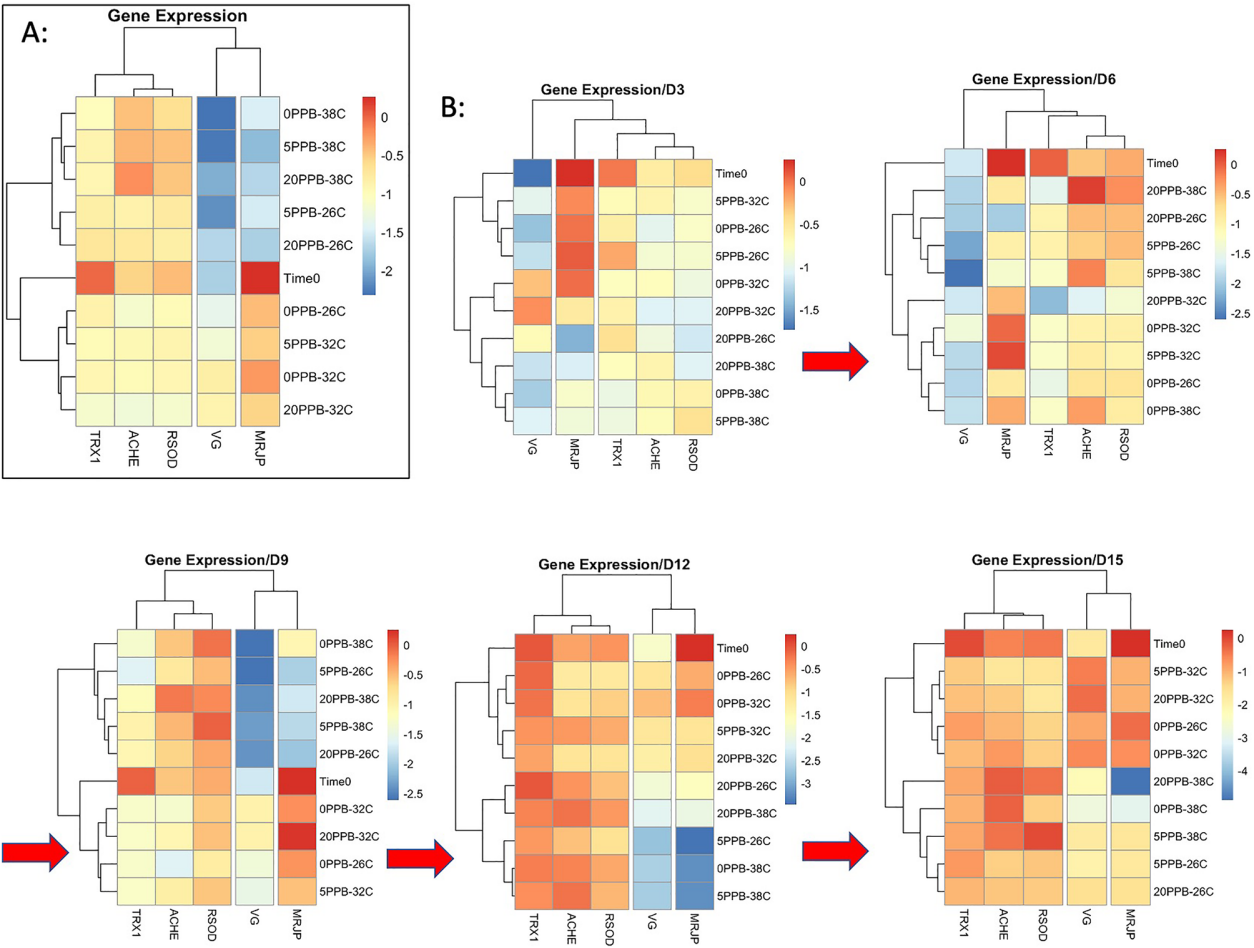
Heatmaps reflecting (**A**) the overall gene expression of all studied genes and treatment, (**B**) longitudinal gene regulations displayed on a day-to-day basis.

The regulation of the studied genes represented by heatmaps largely corroborated the PCA findings, Fig. 11. The heatmap dendrogram of overall gene expression distinguished two main clusters of genes; (*Vg*, *mrjp1*) and (*AChE-2*, *Rsod*, *Trx-1*), and three different treatments; (26 °C, 38 °C), (32 °C) and (Time 0), Fig. 11A. A single group of control bees kept at 26 °C (0 ppb-26C) deviated from its 26 °C category and clustered with the 32 °C group (Fig. 11A), which also clustered with the 32 °C group in the PCA (Fig. 10A). A stark contrast can be seen in the regulation of *Vg* and *mrjp1* induced by the difference in the rearing temperature. Bees exposed to non-optimal temperatures (26 °C and 38 °C) significantly downregulated these two genes, which are mainly upregulated at the optimal 32 °C, Fig. 11A. Despite visible shifts in the gene regulation throughout the studied dates, the major clustering described in the overall gene regulation remained largely consistent, Fig. 11B.

## Discussion

It is difficult to discern the intertwining relationship between environmental factors and honey bee gene regulation, particularly under hive conditions. This cage study greatly helped isolate selected factors and link them to specific gene activity in honey bees. In worker bees, behavioral maturation, task distribution and seasonal phenotypes are mainly governed by gene regulation^35,36^, which in turn responds to outside stressors to maintain honey bee homeostasis. Honey bee colonies maintain brood nest temperatures of 33–35 °C^31,32^, whereas the temperature in the peripheral areas is more prone to fluctuations^37^. The temperature range of the colony cavity in which adult bees operate is usually between 31 and 32 °C^38^. From a toxicological standpoint, post-ingestive aversion responses vis-à-vis imidacloprid, previously described in other invertebrates^39^, were exclusively manifested in this experiment at 26 °C and 32 °C. Bees avoided imidacloprid-laced syrup at these two ambient temperatures and consumed significantly more control syrup, Figs. 2 and 3. This specific diet preference was previously recorded in the case of newly emerged bees^40^ and in summer bees that emerged in the lab^12^. However, winter bees exhibited the opposite diet behavior in a previous dual-choice experiment^12^ and foragers did not avoid nectar-relevant concentrations of imidacloprid^41^, which indicates that such diet behavior is age-related. Interestingly, such avoidance of imidacloprid was absent at the highest temperature category (38 °C), in which average syrup consumption per bee was similar across treatment groups, Fig. 4.

In terms of mortality, our results established statistically significant causality between ambient temperatures and imidacloprid toxicity. Both non-optimal temperatures (26 °C, 38 °C) significantly amplified the toxicological effect of the imidacloprid according to the Kaplan–Meier models, Figs. 2 and 4. This effect was not recorded (*P* = 0.3) at the optimal temperature of 32 °C, Fig. 3. Similar results were obtained on young bees of unknown age in a four-day treatment utilizing much higher imidacloprid concentrations (0.25 ppm), in which significantly higher mortality were recorded at 24 °C compared to 35 °C^42^. Since there is no evidence to support the assumption of higher imidacloprid intake at 26 °C and 38 °C compared to 32 °C (Fig. S1), it can be proposed that caged bees subjected to lower and higher temperatures may have a deficiency in the detoxification process. This is particularly plausible knowing that the closest overall transcription profiles of detoxification-related genes to field bees were seen in caged bees raised at 30 °C^43^.

This theory is well supported by the regulation of the studied genes except for *Trx-1*, which showed no response to changes in ambient temperatures (Fig. 9) or imidacloprid exposure, Fig. S6. *Trx-1,* with predicted mitochondrial localization*,* is a member of the Thioredoxins family (TRXs), which are highly conserved oxidoreductase proteins required to maintain redox homeostasis in the cell^15,44^. *Trx-1* only differed in expression by age in this study, with significantly higher expression in one-day-old bees (T_0_) compared to older ages, Fig. 9. The function of the *Rsod* gene in insects remains unclear. This gene has been described as an atypical member of the superoxide dismutase family (SOD), which protects against reactive oxygen species (ROS) produced in the mitochondria by converting superoxide to oxygen and hydrogen peroxide^15^. Recent studies have shown that *Rsod* is widely distributed and diverse in the animal kingdom, with activity in multiple cell compartments^45^. Our results show that *Rsod* and *AChE-2* were constantly upregulated at 38 °C but not at 26 °C, regardless of imidacloprid treatment, Figs. 7 and 8. Upregulation of *Rsod* has been previously linked to caging stress^9^ and *AChE-2* to neonicotinoid toxicity^6,28^. This study is the first to report upregulation of *Rsod* and *AChE-2* under controlled and consistent heat stress in cage settings. Expression of *AChE-1*, a homolog of *AChE-2* with much lower catalytic activity toward acetylcholine, was clearly induced by a heat shock of 40 °C for 24 h in nurse bees compared to the control at 32 °C^46^. Furthermore, the regulation of *AChE-2* was significantly higher in bees exposed to imidacloprid compared to the control group at 26 °C, Fig. S4. No differences in the *AChE-2* regulation were observed vis-à-vis imidacloprid at other temperatures, which underscores the strong link between ambient temperature and imidacloprid toxicity seen in the Kaplan Meier prediction at 26 °C, Fig. 2.

Honey bee vitellogenin is synthesized in the abdominal fat body cells and released into the hemolymph^47^. This protein promotes honey bee longevity, particularly in queen bees, by acting as an antioxidant and serves an important role in immunity^18,48^. The diet of honey bees has a major implication on *Vg* expression. A recent study showed that pollen-restricted bees had significantly lower *Vg* expression compared to non-restricted bees^49^. This glycolipoprotein, which has been described as more abundant in nurse bees than foragers^50–52^, was the most informative among the other four genes studied. *Vg* appears to be a useful marker for heat stress in honey bees, as it was consistently downregulated at non-optimal temperatures (26 °C, 38 °C) regardless of imidacloprid exposure, Fig. 5. A single instance in which imidacloprid significantly (*P* < 0.05) downregulated *Vg* expression compared to the control was recorded at 5 ppb in the 26 °C group, Fig. S2. However, such downregulation should be carefully considered as it was not recorded within the 20 ppb treatment. Similarly, but to a lesser extent, *mrjp1* followed the trends of *Vg* (Fig. 6), indicating ambient temperature has a profound impact on the regulation of these two genes. The honey bee (*mrjp1*) gene belongs to the major royal jelly protein (MRJP) gene family, which encodes nine major royal jelly protein genes (*mrjp1* to *mrjp9*), *mrjp1* being the most abundant^53–55^. Royal jelly exhibits antioxidant activity likely derived from its MRJPs and peptides^19^. The common expression patterns of *Vg* and *mrjp1* are evident in the overall heatmap results, which show downregulation of both genes at 26 °C and 38 °C compared to other genes. The divergence in their regulation compared to other genes (*Rsod*, *AChE-2*, *Trx-1*) is confirmed by the opposite arrow direction in the PCA for variables, Fig. 10D. The overarching pattern of gene regulation reveals three main clusters from the dimensional projection of individuals (Dim1: 61.1%), which assigned temperature as a major effect, irrespective of the exposure to imidacloprid, followed by the bee age, Fig. 10. A high and healthy expression of both *Vg* and *mrjp* is necessary for worker bees, particularly nurses, to provide care for young larvae and queens. Our data indicates that the highest expressions of both genes occur at the optimal temperature of 32 °C. Interestingly, a study by Kim et al. showed that both *Vg* and *mrjp-1* were significantly induced in nurses upon ingesting live and heat-killed *Paenibacillus larvae*^56^. A previous study on *Drosophila melanogaster* associated MRJP diet supplementation with the upregulation of superoxide dismutase and lifespan extension caused by MRJPs acting as antioxidants in intercellular cytoplasmic compartments^57^. Similar to *Vg*, *mrjp1* has been shown to decrease in nurse bees deprived of pollen^58^, and malnutrition has been shown to decrease *Vg* protein levels, leading to an increase in overwintering mortalities^59^. *Vg* expression, however, was described to decline over the winter in wintering bees^60^. Taken together, low or non-optimal expressions of *Vg* and *mrjp1* could lead to reduced immune responses, and ambient temperature is an additional factor this study has added.

In conclusion, our study provides new evidence of the impact of ambient temperature on imidacloprid toxicity in honey bees. We demonstated that honey bees are significantly more susceptible to imidacloprid at non-optimal ambient temperatures. These findings have significant implications for honey bee colonies during prolonged heat stress and exposure to imidacloprid. The results of this study evidenced strong links between the regulation of major honey bee genes, ambient temperature and exposure to imidacloprid.

## Methods

### Honey bee colony

A single healthy and well-established honey bee colony, headed by a Carniolan *Apis mellifera carnica* queen, was used as a source for all worker samples of this study. Using sister bees originating from a single colony helps minimize variability in hive conditions and the genetic make-up of the workers. A total of eight capped worker brood frames ready to hatch were removed from this hive and placed in an incubator at 35 °C with 50–60% relative humidity. The following day, several thousand one-day-old sister bees were collected into a sterile plastic box for further use.

### Cage experiment

Newly emerged worker bees collected in the plastic box were gently mixed and distributed into 27 cages with an average of 100 bees per cage. The 27 bee cages were given a 2-day acclimatization period in which they were stored at 32 °C (55% RH) and supplied with a clean 1:1 sugar syrup solution *ad libitum*. Subsequently, at day 0 the treatments were applied, and cages were randomly assigned to two imidacloprid toxicity groups and a control (0 ppb, 5 ppb, 20 ppb). Cages were randomly divided into three groups, each subjected to a different temperature (26 °C, 32 °C, 38 °C), Fig. 1. Bees in each cage were provided with sugar syrup (1:1) using 20-mL syringes and 10 g of Global Protein Patty, which consisted of 15% pollen, sugar, soy flour, brewer’s yeast and water (BetterBee Co., NY, USA) placed into rubber plugs. Imidacloprid was administrated to bees through the sugar syrup at the concentrations mentioned above. Patty and syrup consumptions were recorded daily by weight difference using a ± 0.01 g sensitive scale. Dead bees were counted daily and cleared from the cages.

### Honey bee sampling

Worker bees were sampled at five time points for molecular analysis. Five workers per cage were sampled on days 3, 6, 9, 12 and 15 of the experiment. Additionally, approximately one hundred one-day old bees were randomly collected at the beginning of the experiment right after emergence (Day -2) to be used as the Time 0 Reference. All sampled bees were euthanized by placing them on dry ice and stored at –80 °C for subsequent molecular analyses.

### RNA extraction

RNA was extracted from whole bee bodies in an RNase-free, sterile work environment. Total RNA was extracted from a pool of 3 bees per sample using TRIzol® Reagent protocol from Invitrogen^61^ coupled with Zymo RNA Extraction Kit. Three bees were subsampled from dry ice for each collection and immediately placed in a 2 mL screw cap tube with 1 mL TRIzol and 5 mg of acid-washed glass beads. Bee samples were homogenized for 2 min (4 × 30 s) using a FastPrep MP Biomedicals homogenizer (Irvine, CA, USA). Three hundred microliters of the homogenate was transferred to a fresh 1.5 mL tube and mixed thoroughly with 300 µL of 95% Ethanol. The total 600 µL product was transferred to a Zymo-Spin Column and centrifuged at 10,000 g for 2 min. DNase treatment, RNA purification and precipitation were carried out according to the manufacturer’s protocol. Final RNA extractions were nanodropped (Thermo Scientific NanoDrop 2000/2000c Spectrophotometers) for RNA quantity and quality. RNA extractions were diluted to 200 ng/μL and stored at − 80 °C.

### Transcriptional analysis

A total of five genes were investigated in this study, namely: 1- vitellogenin (*Vg*), 2- major royal jelly protein 1 (*mrjp1*), 3- acetylcholine esterase 2 (*AChE-2*), 4- superoxide dismutase-like (*Rsod*) and 5- thioredoxin 1 (*Trx-1*). Sequences of the primers and their amplicon sizes are given in Table 1. Two-step reverse transcription quantitative PCR (RT-qPCR) using BioRad iTaq SYBER Green Supermix 2X was conducted on three biological and technical replicates per sample on five time points enabling a greater longitudinal analysis of gene regulation. cDNA was synthesized from RNA extractions using BioRad iScript Kit following the manufacturer’s protocol. Target genes were normalized against two housekeeping genes (*GAPDH*, *RPS18*) known for their stability in honey bee tissues^38,62^.

**Table 1:** Target genes investigated in this study, housekeeping genes, primer sequences, amplicon size and NCBI accession numbers.

Gene	Description	F/R	bp	NCBI Accession
Target				
*AChE-2*	Acetylcholinesterase-2	GACGCGAAGACCATATCCGT TCTGTGTCCTTGAAGTCCGC	140	NM_001040230.1
*Mrjp1*	Major royal jelly protein 1	TGACCAATGGCATGATAAG GACCACCATCACCGACCT	98	NM_001011579.1
*Vg*	Vitellogenin	AACGCTTTTACTGTTCGCGG TATGCACGTCCGACAGATCG	128	NM_001011578.1
*Rsod*	Superoxide dismutase-like	GGAGCAGTATCTGCAATGGGA CGCTACAAAACGTGGTGGTT	141	XM_006558333.2
*Trx-1*	Thioredoxin-1	AATGCACCGGCTCAAGAACA CATGCGACAAGGATTGCACC	138	XM_393603.7
Housekeeping				
*GAPDH*	Glyceraldehyde-3-phosphate dehydrogenase 2	TACCGCTTTCTGCCCTTCAA GCACCGAACTCAATGGAAGC	142	XM_393605.7
*RPS18*	40S ribosomal protein S18	AATTATTTGGTCGCTGGAATTG TAACGTCCAGCAGAATGTGGTA	238	XM_625101.6

### Data analysis

Statistical analyses were carried out in the R environment^63^ via RStudio Version (2022.07.0). This study was conducted at the cage level with three biological replicates and three variables: syrup consumption, patty intake and bee mortality. The regulation of five different genes was evaluated using three biological and technical replicates per sample. Each studied sample represents a transcriptional RNA pool originating from three different bees. All datasets were tested for normality using the Shapiro test. ANOVA was conducted at a 95% confidential interval with three levels of significance (*P* < 0.05, < 0.001, < 0.001) on data normally distributed. Kruskal–Wallis rank test, a non-parametric test, was used on data that failed the normality test in which multiple comparisons and p-values were adjusted with the Benjamini–Hochberg method. Syrup and patty consumptions were calculated at bee level (g/bee) by dividing the cage’s daily consumption by the number of bees alive at the time of the reads. Survival probability and cumulative hazard were calculated for each temperature group by the Kaplan–Meier survival probability model in R using three Packages: “dplyr”, “survival”, “survminer”. Figures were generated in the same environment utilizing four main libraries: “ggplot2”, “doby”, “plyr”, and “beeswarm”. Gene regulations were displayed longitudinally and as overall averages, either by temperature or treatment effects over time. Principal component analysis (PCA) was conducted using the overall average expressions of the five studied genes. PCA was carried out using the “factoextra” library to estimate the expression of each variable on a 3-dimensional scale and treatment group similarity. Heatmaps were generated using the “pheatmap” library either by sampled dates (5 dates) or by the overall average expression of each gene similar to the PCA. All error bars of this study represent the Standard Error (SE) except for the boxplots (box and whisker plots), which display the median, first and third quartiles, and both maximum and minimum values of variables.

## Supplementary Information

Supplementary Information.

## Data Availability

All data related to this study are included in this manuscript and its supplementary materials.
