# Supplementary material for: Non-optimal ambient temperatures aggravate insecticide toxicity and affect honey bees Apis mellifera L. gene regulation
Source: Sci Rep. 2023 Mar 9;13:3931. doi: 10.1038/s41598-023-30264-0 (PMC9998868; doi:10.1038/s41598-023-30264-0)

**Figure S1.** Overall average syrup and patty consumption across imidacloprid and temperature treatments. Bee group at 32°C consumed significantly higher syrup than the 26°C and 38°C groups. The bee group at 38°C consumed significantly less protein than both groups.


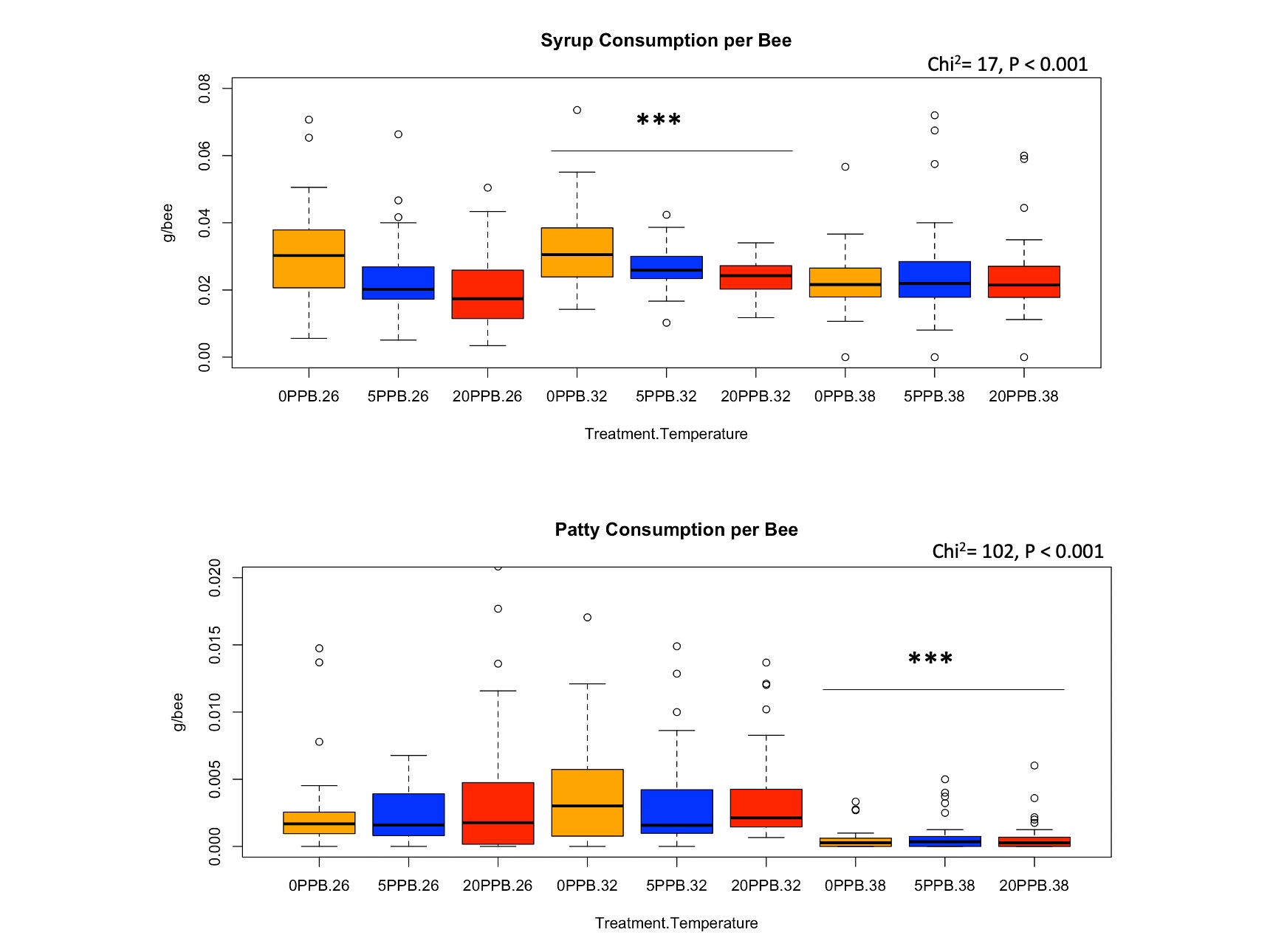


**Figure S2.** Gene expression of *vg* displayed by day and overall average for imidacloprid treatments (0 PPB, 5 PPB, 20 PPB) in each temperature’s categories (26°C, 32°C, 38°C). Error bars of the line graphs represent the Standard Error SE.


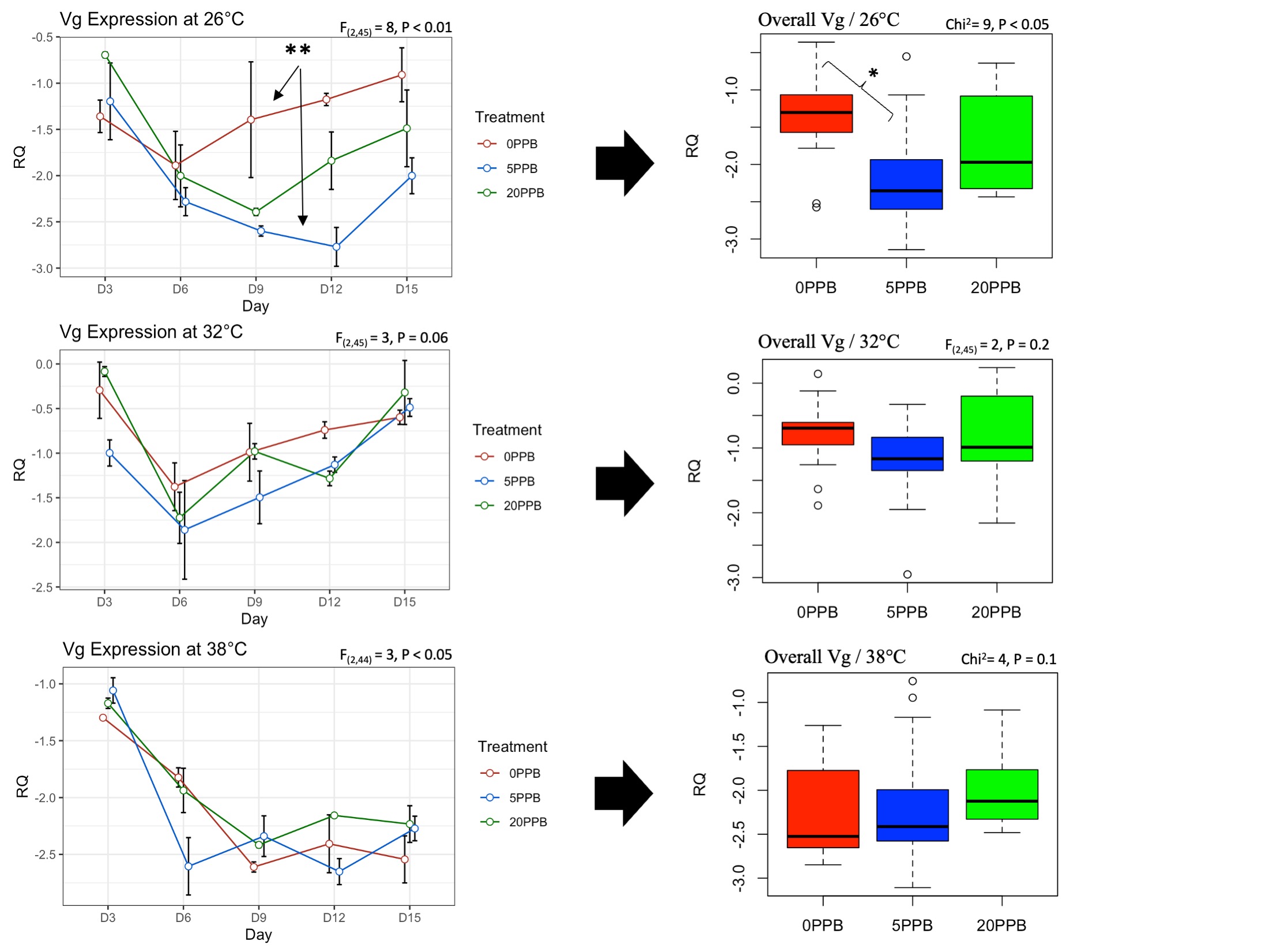


**Figure S3.** Gene expression of *mrjp1* displayed by day and overall average for imidacloprid treatments (0 PPB, 5 PPB, 20 PPB) in each temperature’s categories (26°C, 32°C, 38°C). Error bars of the line graphs represent the Standard Error SE.


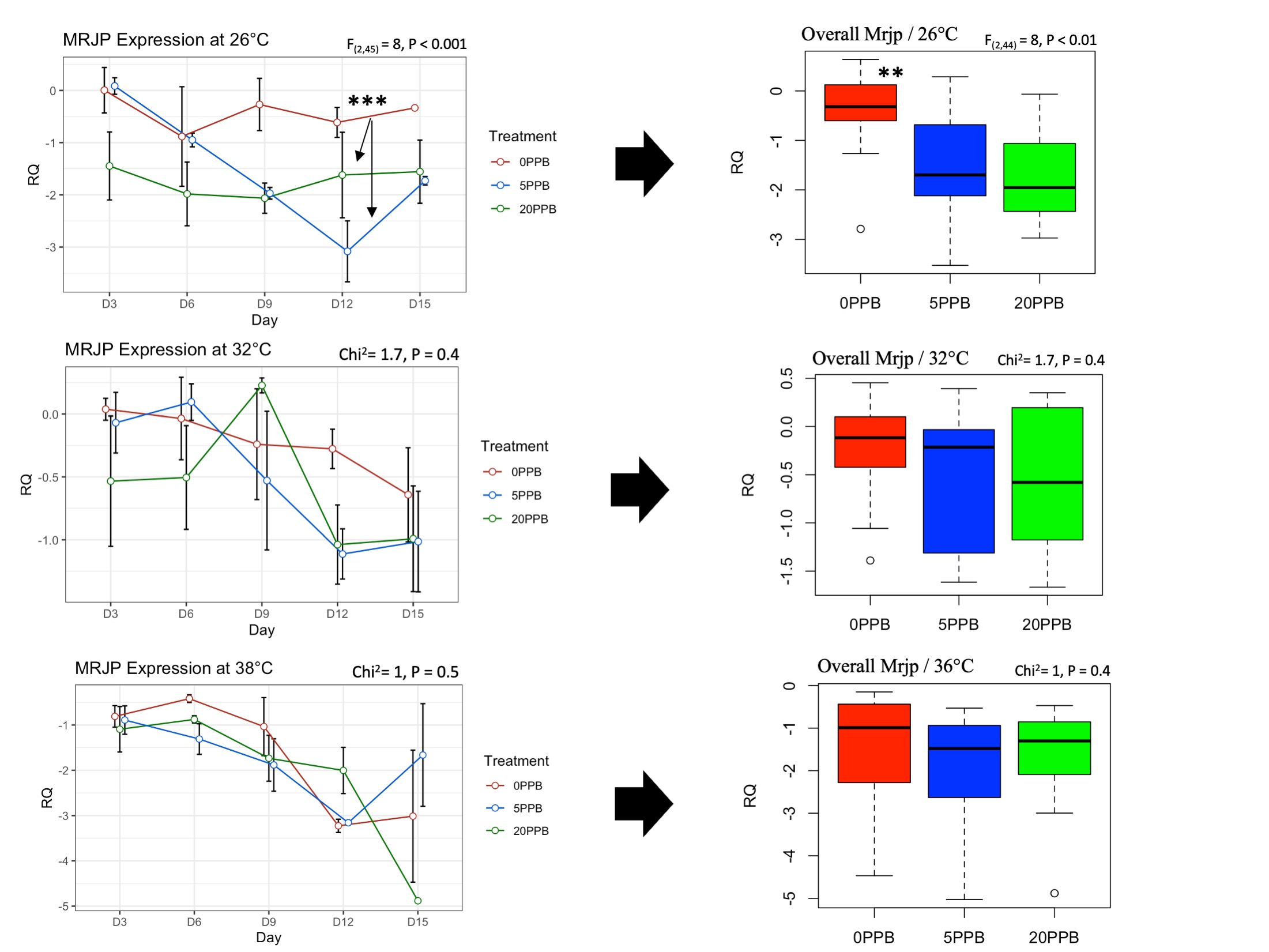


**Figure S4.** Gene expression of *AChE-2* displayed by day and overall average for imidacloprid treatments (0 PPB, 5 PPB, 20 PPB) in each temperature’s categories (26°C, 32°C, 38°C). Error bars of the line graphs represent the Standard Error SE.


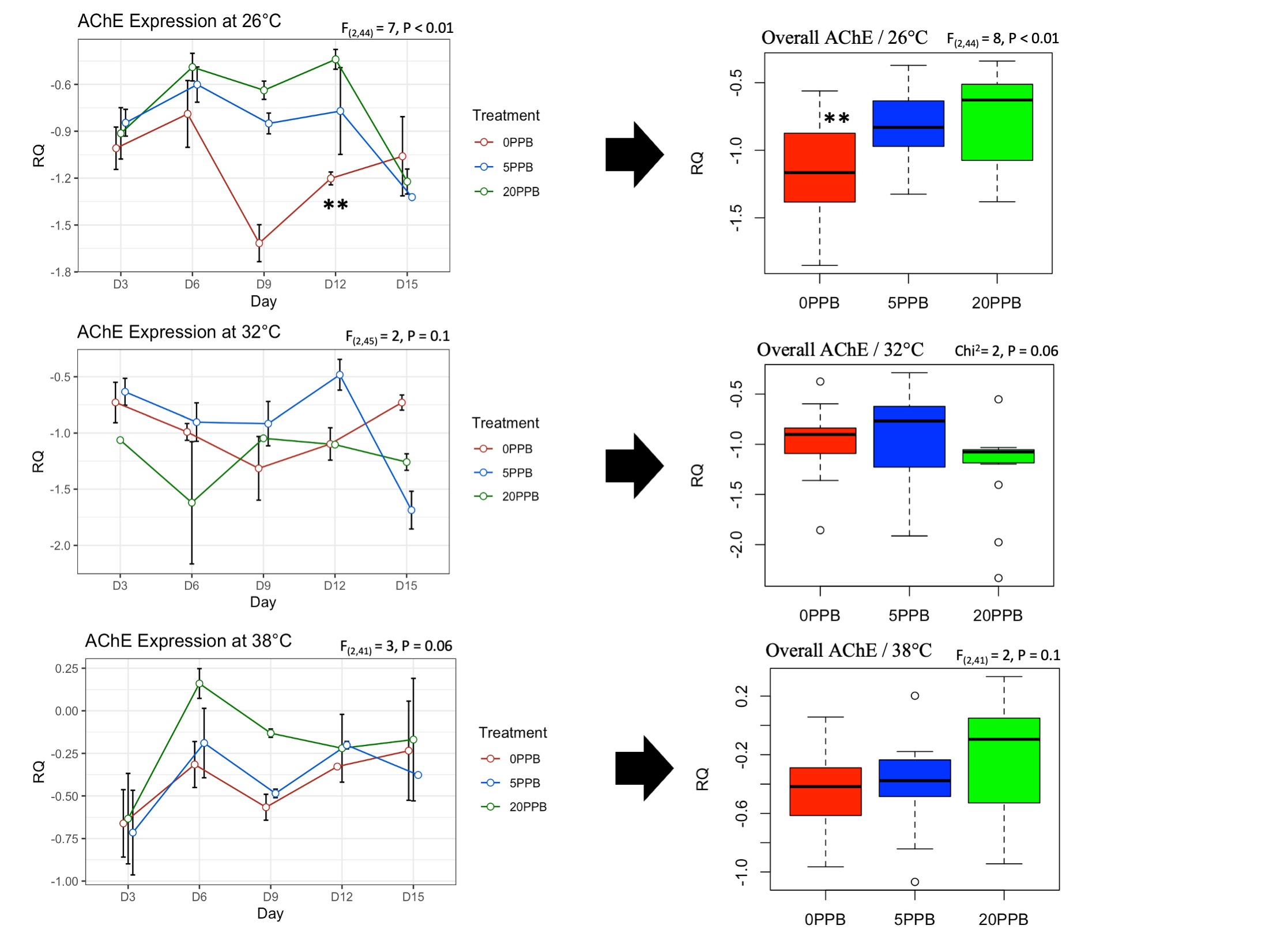


**Figure S5.** Gene expression of *Rsod* displayed by day and overall average for imidacloprid treatments (0 PPB, 5 PPB, 20 PPB) in each temperature’s categories (26°C, 32°C, 38°C). Error bars of the line graphs represent the Standard Error SE.


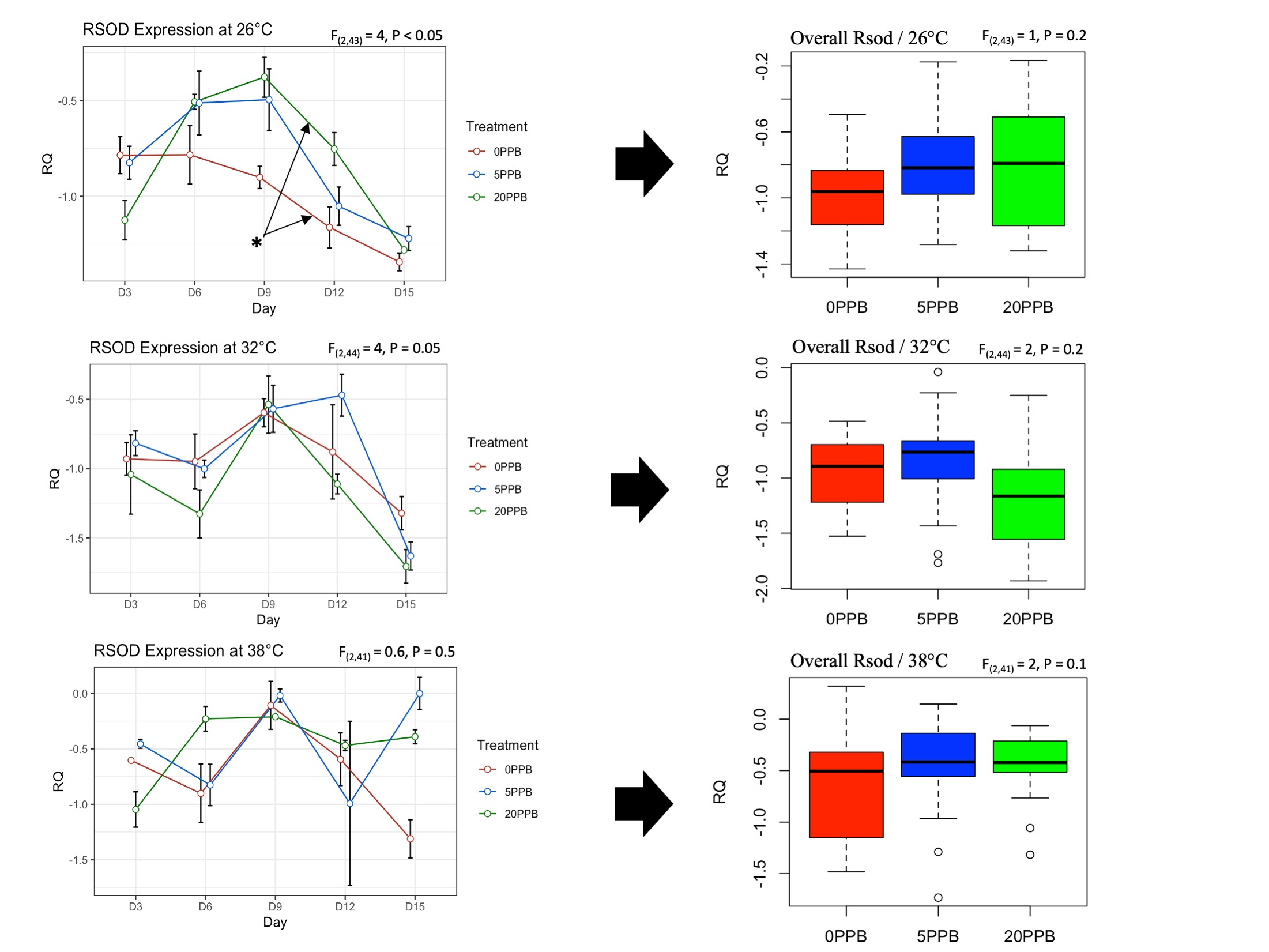


**Figure S6.** Gene expression of *Trx-1* displayed by day and overall average for imidacloprid treatments (0 PPB, 5 PPB, 20 PPB) in each temperature’s categories (26°C, 32°C, 38°C). Error bars of the line graphs represent the Standard Error SE.


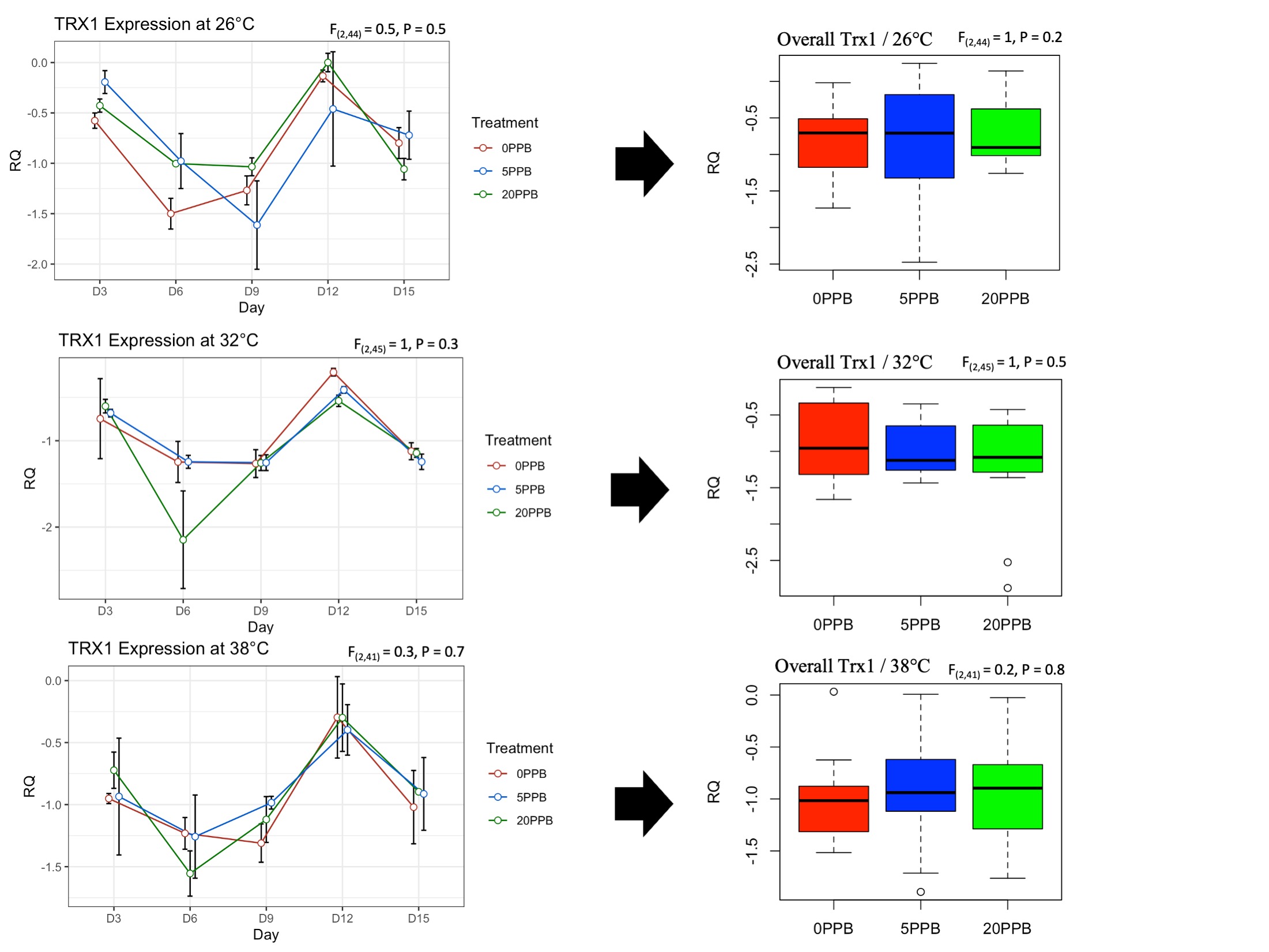

Supplement: Supplementary file 1 — Supplementary Information. [file 41598_2023_30264_MOESM1_ESM.docx]
